# CT-Referenced Diagnostic Performance and Complementary Error Patterns of Artificial Intelligence Versus a Single Emergency Physician in Chest Radiograph Interpretation: A Retrospective Paired Diagnostic Accuracy Study

**DOI:** 10.3390/tomography12100142

**Published:** 2026-10-01

**Authors:** Ömer Damar, Mahmut Yaman

**Affiliations:** 1 Department of Emergency Medicine, Faculty of Medicine, Mardin Artuklu University, 47200 Mardin, Türkiye; 2 Department of Emergency Medicine, Faculty of Medicine, Dicle University, 21280 Diyarbakır, Türkiye

**Keywords:** artificial intelligence, chest radiography, diagnostic accuracy, emergency medicine, human-AI complementarity, clinical decision support

## Abstract

**Background/Objectives:** Artificial intelligence (AI) is increasingly used to support chest radiograph interpretation, but its clinical value depends not only on stand-alone accuracy but also on whether its errors differ from those of clinicians. We evaluated CT-referenced, target-specific diagnostic performance and complementary error patterns of a commercial chest radiograph AI system and a single emergency physician. **Methods:** In this retrospective, single-center, paired diagnostic accuracy study, 920 consecutive adults who underwent posteroanterior chest radiography and thoracic computed tomography (CT) within 4 h during the same emergency department encounter were included. A commercial AI system (hChestXR version 1) and a single blinded emergency physician independently assessed eight prespecified thoracic findings using CT as the reference standard. Paired differences were estimated with 10,000 patient-level bootstrap resamples and tested with exact McNemar tests with Holm adjustment. Exact-target complementarity and simulated same-target OR/AND rules were secondary exploratory analyses. **Results:** Across 7360 patient–target assessments, including 1232 CT-positive findings, AI had significantly greater Holm-adjusted sensitivity for pneumothorax (90.8% vs. 50.8%), nodule/mass (63.3% vs. 32.7%), and rib fracture (77.2% vs. 46.5%). AI-only detections were most frequent for pneumothorax (47.7%), nodule/mass (44.9%), and rib fracture (43.6%), whereas physician-only detections were most frequent for atelectasis (24.8%), pulmonary edema (21.0%), and lung opacity (19.7%). In exploratory pooled analyses, sensitivity was 73.0% for AI and 62.0% for the physician. Exploratory simulated same-target OR/AND analyses demonstrated a sensitivity–specificity trade-off across pooled patient–target assessments but did not represent observed AI-assisted clinical performance. **Conclusions:** The AI system and the single emergency-physician reader in the study showed target-dependent, partly non-overlapping error patterns in this CT-selected emergency cohort. The findings support prospective multireader evaluation of AI as a second-reader tool but do not establish benefit from real-time AI-assisted interpretation.

## Simple Summary

Artificial intelligence may help clinicians detect abnormalities on chest radiographs, but its usefulness depends on whether it identifies findings that clinicians miss. In 920 emergency department patients who underwent chest radiography and chest computed tomography within four hours, we compared a commercial artificial intelligence system with a single emergency physician using computed tomography as the reference. The two readers showed partly different error patterns. Combining their independent findings increased sensitivity but reduced specificity. Prospective multireader studies are needed to determine whether displaying artificial intelligence results improves real-time clinical decisions.

## 1. Introduction

Chest radiography remains one of the first-line imaging examinations for patients presenting to emergency departments with dyspnea, chest pain, trauma, or suspected infection because it is rapidly obtainable, widely available, relatively inexpensive, and associated with a limited radiation dose [1]. Nevertheless, chest radiograph interpretation is intrinsically difficult. Multiple anatomical structures are superimposed on a two-dimensional image; findings such as a small pneumothorax, pulmonary nodule, rib fracture, or early parenchymal opacity may be subtle; and reader performance can be affected by experience and clinical workload. In a study comparing emergency physician interpretations with those of senior radiologists, sensitivity for individual radiographic abnormalities ranged from 20% to 64.9%, while overall interobserver agreement remained moderate [2]. The limitations of radiography are not attributable solely to the reader. A multicenter emergency department study that compared chest radiographs with chest CT examinations obtained in the same patients reported a sensitivity of 43.5% and a specificity of 93.0% for the radiographic detection of pulmonary opacities, demonstrating substantial discordance between the two modalities [3]. Thus, although chest radiography occupies a central position in acute care, accurate interpretation and verification against a more sensitive anatomical reference remain important components of diagnostic accuracy research.

Advances in deep learning–based image analysis have extended the scope of automated chest radiograph interpretation from single-disease screening to the simultaneous detection of multiple thoracic abnormalities. The CheXNeXt algorithm evaluated 14 pathologies concurrently and achieved performance comparable to that of radiologists for several findings, while also demonstrating that performance varied according to the target abnormality [4]. Another algorithm, validated across five independent datasets, showed high classification and localization performance for pulmonary malignant neoplasm, active tuberculosis, pneumonia, and pneumothorax; its use as a decision-support tool improved the performance of physicians with different levels of expertise [5]. More broadly, the object-detection methods underlying such AI-generated localization outputs have evolved from classical handcrafted-feature pipelines toward end-to-end convolutional detectors, a transition that parallels developments across computer vision generally [6]. In an emergency department validation study, a deep learning algorithm detected clinically relevant thoracic abnormalities and increased the sensitivity of radiology residents after its outputs were made available [1]. Automated prioritization represents an additional application: a simulation study showed that AI-based triage could reduce reporting delays for radiographs containing critical or urgent findings [7]. In a multireader study covering 127 clinical findings, deep learning assistance increased radiologists’ macroaveraged AUC from 0.713 to 0.808 and significantly improved classification accuracy for 102 findings [8]. Similarly, a reader study involving 758 radiographs and five radiologists with different experience levels found that AI assistance increased sensitivity and reduced interpretation time [9]. A recent evaluation of commercial AI in chest radiographs referred from an emergency department further reported that diagnostic performance differed across fractures, pneumothorax, pulmonary opacities, and pleural effusions [10]. This heterogeneity supports evaluating each target as a separate binary diagnostic task rather than relying exclusively on a global “abnormal radiograph” endpoint. It also highlights the value of exact matching, in which each AI and physician classification is compared only with the corresponding reference-standard finding. Consistent with this approach, the STARD-AI guideline recommends transparent reporting of dataset construction, the AI index test, the reference standard, and the procedures used to evaluate diagnostic performance [11].

The clinically relevant question is not only whether an AI system outperforms a physician, but whether the two readers make sufficiently different errors to provide complementary diagnostic information. We therefore evaluated a commercial PACS-integrated AI system and one blinded emergency physician in adult emergency department patients with temporally proximate posteroanterior chest radiography and thoracic CT. The primary objective was to compare CT-referenced, target-specific sensitivity and specificity across eight prespecified thoracic findings. Secondary objectives were to quantify paired performance differences, characterize complementary error patterns through non-overlapping detections, and explore the sensitivity–specificity trade-offs of same-target OR and AND rules. These combination rules were prespecified simulations of independent readings and were not interpreted as observed real-time AI-assisted physician performance.

## 2. Materials and Methods

### 2.1. Study Design, Setting and Ethics

This retrospective, single-center, paired diagnostic accuracy study was conducted in the adult emergency department of Mardin Training and Research Hospital, a tertiary-care teaching hospital in Türkiye, using eligible clinical encounters occurring between 1 March 2025 and 28 February 2026. The performance of a commercial artificial intelligence (AI) system and a single blinded emergency physician was compared with thoracic computed tomography (CT) as the reference standard. The study is reported in accordance with STARD-AI [11]. The institutional ethics committee approved the protocol (14 July 2026; meeting no. 07; decision no. 20; protocol no. 2026/07-20) and waived written informed consent because the study was retrospective and used anonymized data. The study complied with the Declaration of Helsinki. Although eligible clinical encounters spanned 1 March 2025–28 February 2026, before this ethics-approval date, all research-specific procedures—including dataset extraction, the blinded physician re-interpretation described below, CT-target adjudication, and statistical analysis—were conducted only after ethics approval, using retrospectively anonymized data; no encounter-level research activity preceded approval.

### 2.2. Participants and Image Acquisition

**Figure 1:**
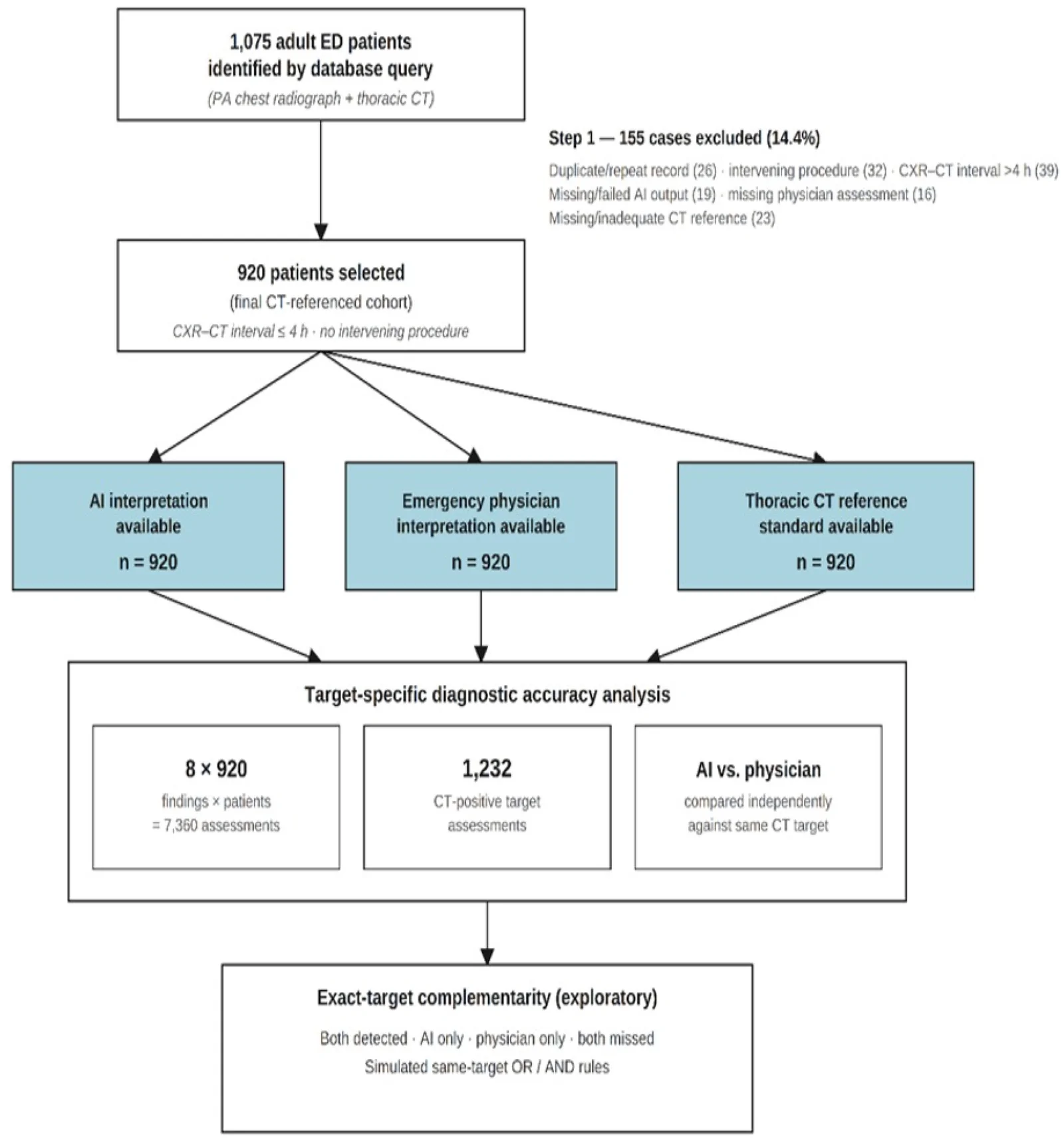
STARD-AI participant flow diagram. The final cohort comprised 920 patients with complete AI, emergency physician, and CT reference-standard data. The primary analysis included 7360 patient–target assessments across eight prespecified findings; exact-target complementarity and the same-target OR/AND rules were exploratory analyses.

The institutional PACS and hospital information system were queried for all consecutive adults (≥18 years) who underwent posteroanterior chest radiography (CXR) and thoracic CT during the same emergency department encounter. Eligible patients had a CXR-to-CT interval of ≤4 h and complete AI, emergency-physician, and CT assessments. Only the first eligible encounter per patient was included; when multiple radiographs were available, the first eligible posteroanterior image obtained before CT was selected. Portable anteroposterior and lateral radiographs were excluded. Other exclusions were duplicate records, a CXR-to-CT interval > 4 h, failed AI processing or transmission, unavailable physician assessment, and missing, inaccessible, technically inadequate, or indeterminate CT reference data. Patients undergoing an image-altering intervention between examinations, including tube thoracostomy, endotracheal intubation, or thoracentesis, were also excluded. No sampling was performed, and each included patient contributed one index radiograph and eight target assessments. Patient flow through screening, exclusion, and inclusion is summarized in Figure 1.

Posteroanterior radiographs were acquired using a GC80CW digital radiography system (Samsung Electronics Co., Ltd., Suwon, Republic of Korea) according to routine departmental protocols. Thoracic CT was performed using an Alexion multidetector scanner (Toshiba Medical Systems Corporation, currently Canon Medical Systems Corporation, Otawara, Japan) at 80–135 kVp and 10–300 mA, with a 512 × 512 matrix and 0.5–5 mm reconstructions according to clinical indication. Contrast-enhanced, non-contrast, and CT pulmonary angiography examinations were eligible. Acquisition timestamps recorded in the PACS were used to calculate the CXR-to-CT interval; the first CT obtained after the index radiograph within 4 h served as the reference examination.

### 2.3. Artificial Intelligence System

The index AI test was hChestXR version 1 (Hevi AI, Istanbul, Türkiye), which was integrated into the hospital PACS and used in routine clinical practice. Radiographs were processed automatically in real time and were not retrospectively reprocessed. The system evaluated eight prespecified targets: pleural effusion, pneumothorax, consolidation/infiltration, atelectasis, nodule/mass, pulmonary edema, lung opacity, and fracture. A separate 1–100 confidence score was generated for each target, with localization overlays displayed for positive findings. The manufacturer-defined threshold was used without local modification: scores > 50 were positive and scores ≤ 50 were negative. The software version and threshold remained unchanged during the study period. This threshold was fixed by the manufacturer prior to the experiment and independently of this study population; it was not tuned or optimized using any of the 920 patients analyzed here, so the reported estimates are not subject to in-sample threshold-optimization bias.

Because numerical scores ≤ 50 were not accessible, AI output was analyzed only at the predefined binary operating point; ROC, calibration, and alternative-threshold analyses were therefore not performed. The “No Findings” output represented absence of all eight targets and was not analyzed separately. AI turnaround time was calculated from CXR acquisition to AI report timestamps in the PACS.

### 2.4. Blinded Emergency Physician Interpretation

All index radiographs were evaluated once by an emergency-medicine specialist with 10 years of experience using the hospital PACS and a standard diagnostic workstation. Images were anonymized, assigned random codes, and presented in random order. The physician had access only to the index radiograph and was blinded to patient demographics, clinical and laboratory data, previous imaging, AI outputs, radiology reports, and CT findings. Routine viewing functions, including magnification and window adjustment, were permitted. Each target was classified independently as present or absent; no indeterminate category was available. This blinded interpretation was a dedicated research assessment performed after institutional ethics approval and was distinct from, and not used in place of, any original real-time clinical impression documented in the emergency department chart at the time of the index encounter. Because the physician was blinded to AI output and both were compared independently against the CT reference standard, this design evaluates independent AI-versus-physician performance rather than AI-assisted interpretation. Because the physician interpreted radiographs without clinical information, laboratory data, or prior imaging, this design isolates image-only interpretive performance; it is therefore a controlled experimental comparison rather than a reconstruction of real-time emergency-department practice, in which physicians integrate radiographs with clinical context.

### 2.5. CT Reference Standard and Target Harmonization

The reference standard was the final signed thoracic CT report issued in routine practice by board-certified radiologists with at least 5 years of experience, who did not have access to the AI output. hChestXR output is displayed only within the emergency-department PACS viewer used by emergency physicians and is not surfaced within the separate radiology-department reporting workstation and report-generation software used by the radiologists who issue thoracic CT reports; issuing a CT report is a radiology-department function performed on this separate system, so reporting radiologists did not encounter hChestXR output in the course of routine CT interpretation for this cohort. Reports with terminology that precluded reliable classification were reassessed by a second radiologist; unresolved cases were excluded. Investigators extracted CT findings using prespecified definitions. Hemothorax was mapped to pleural effusion; pneumonia and pulmonary contusion to consolidation/infiltration; and nodules and masses to a combined nodule/mass target. The AI fracture category was matched to CT-confirmed rib fracture, while lung opacity was coded when explicitly stated in the CT report. Findings described as probable, suspicious, possible, or not excludable were considered positive. Targets were not mutually exclusive. A full target-by-target harmonization table specifying the exact AI label, physician label, CT definition, and accepted synonyms for each of the eight targets is provided in Supplementary Table S3. As specified among the eight prespecified AI targets, one was labeled generically as ‘fracture’ rather than ‘rib fracture’; in this study, it was evaluated exclusively against CT-confirmed rib fracture, and other osseous thoracic injuries were not used as reference-positive events for this target. A total of 9/920 patients (1.0%) were AI-positive for ‘fracture’ without a CT-confirmed rib fracture and were scored as false positives for this target; because non-rib thoracic fractures were not systematically coded in this study, we could not determine how many of these nine represented genuine detections of a different thoracic bone injury rather than true errors, and we have not attempted to quantitatively bound this effect. This target-mapping mismatch is instead acknowledged directly as a limitation. The permissive handling of uncertain CT terminology (‘probable’, ‘suspicious’, ‘possible’, or ‘not excludable’ classified as positive) was adopted to avoid under-ascertainment bias, because such hedged findings commonly still prompt clinical action in routine practice. Certainty language was not retained as a separate variable in our structured extraction, so a sensitivity analysis restricted to definite findings could not be performed in this revision; independent, blinded re-adjudication of uncertain-terminology findings against prespecified definitions is accordingly identified as a specific priority for prospective validation.

Exact target matching was used throughout: each AI and physician classification was compared only with the corresponding CT finding. Detection of a different abnormality was not counted as a true-positive result for the target under evaluation.

### 2.6. Outcomes

The primary outcomes were target-specific sensitivity and specificity for AI and physician interpretations. Secondary measures were accuracy, positive and negative predictive values (PPV and NPV), positive and negative likelihood ratios, F1 score, and Cohen’s kappa. Among CT-positive targets, complementarity was categorized as detected by both readers, AI only, physician only, or missed by both. A same-target OR rule was positive when either reader identified the target, whereas an AND rule required both readers to be positive. These combinations were evaluated in simulated secondary analyses and did not represent prospective AI-assisted rereading. Workflow outcomes were AI turnaround time, CXR-to-CT interval, and CT report turnaround time. We use ‘finding-level’ (or ‘target-level’) to denote the unit of this analysis—one binary presence/absence judgment for one prespecified target in one patient—as distinct from ‘patient-level,’ a single combined judgment of whether a patient has any abnormality; the primary and secondary diagnostic-accuracy analyses in this study are exclusively finding-level. The both-detected/AI-only/physician-only/both-missed categorization above constitutes a four-way paired classification of AI and physician outcomes for each CT-positive target, quantifying not only how often each reader erred but specifically which cases each reader alone detected.

### 2.7. Statistical Analysis

Continuous variables were summarized as mean ± standard deviation or median [interquartile range], as appropriate; categorical variables were reported as n (%). For each target and reader, 2 × 2 tables were used to calculate diagnostic measures. Sensitivity, specificity, accuracy, PPV, and NPV were reported with Wilson 95% confidence intervals (CIs). Because both readers evaluated the same patients, differences in sensitivity, specificity, and accuracy were analyzed as paired percentage-point differences with 10,000 patient-level bootstrap resamples. Exact two-sided McNemar tests—paired tests appropriate for correlated binary outcomes from the same patients—compared sensitivity among CT-positive cases, specificity among CT-negative cases, and accuracy in the full cohort. Holm adjustment was applied separately across the eight targets for each family of comparisons; adjusted *p* < 0.05 was considered significant.

Complementarity proportions used CT-positive cases for the corresponding target as the denominator and were reported with Wilson 95% CIs. The exploratory micro-averaged analysis pooled all patient–target observations; its CIs were obtained using 10,000 patient-cluster bootstrap resamples to preserve within-patient correlation. Target-specific estimates remained primary. As a complementary descriptive analysis, we additionally computed macro-averaged sensitivity, specificity, and accuracy, in which each of the eight targets contributes equally regardless of prevalence (unweighted mean of the eight target-specific estimates), using the same patient-level bootstrap methodology (10,000 resamples). Robustness was assessed in patients with a CXR-to-CT interval ≤ 120 min. No imputation was performed because the final cohort had complete index-test and reference-standard data, and no a priori sample-size calculation was undertaken because all eligible consecutive patients were included. Analyses used Python 3.12.13 with pandas 2.2.3, NumPy 2.3.5, and SciPy 1.17.0. During manuscript preparation, ChatGPT (GPT-5.6; OpenAI, San Francisco, CA, USA) was used for language refinement, statistical coding support, and figure preparation. All AI-assisted outputs were reviewed and verified by the authors, who retain full responsibility for the analysis and final manuscript.

## 3. Results

Among 1075 adult emergency department patients identified during the study period, 155 were excluded because of duplicate/repeat records (n = 26), intervening treatment (n = 32), a CXR-to-CT interval > 4 h (n = 39), missing/failed AI output (n = 19), missing physician assessment (n = 16), or missing/inadequate CT reference (n = 23). The final CT-referenced cohort therefore comprised 920 patients (one index radiograph per patient), all of whom had complete AI, emergency physician, and CT reference-standard data. Across eight prespecified target findings, this yielded 7360 patient–target assessments, including 1232 CT-positive assessments (Figure 1).

The mean age was 55.0 ± 18.4 years, and 479 patients (52.1%) were male. Trauma (29.7%), dyspnea (25.9%), and chest pain (17.2%) were the most frequent presenting complaints. Most patients were assigned to the yellow triage category (55.4%), while 19.3% were categorized as red. The median emergency department length of stay was 242 min [IQR, 154–331.5]. Median AI report turnaround was 4 min [IQR, 3–6], the median CXR-to-CT interval was 73 min [IQR, 51–100], and median CT report turnaround was 146 min [IQR, 116–193]. Overall, 689 patients (74.9%) had at least one CT-confirmed target finding, 282 (30.7%) were admitted to a ward, and 99 (10.8%) required intensive care admission (Table 1).

**Figure 2:**
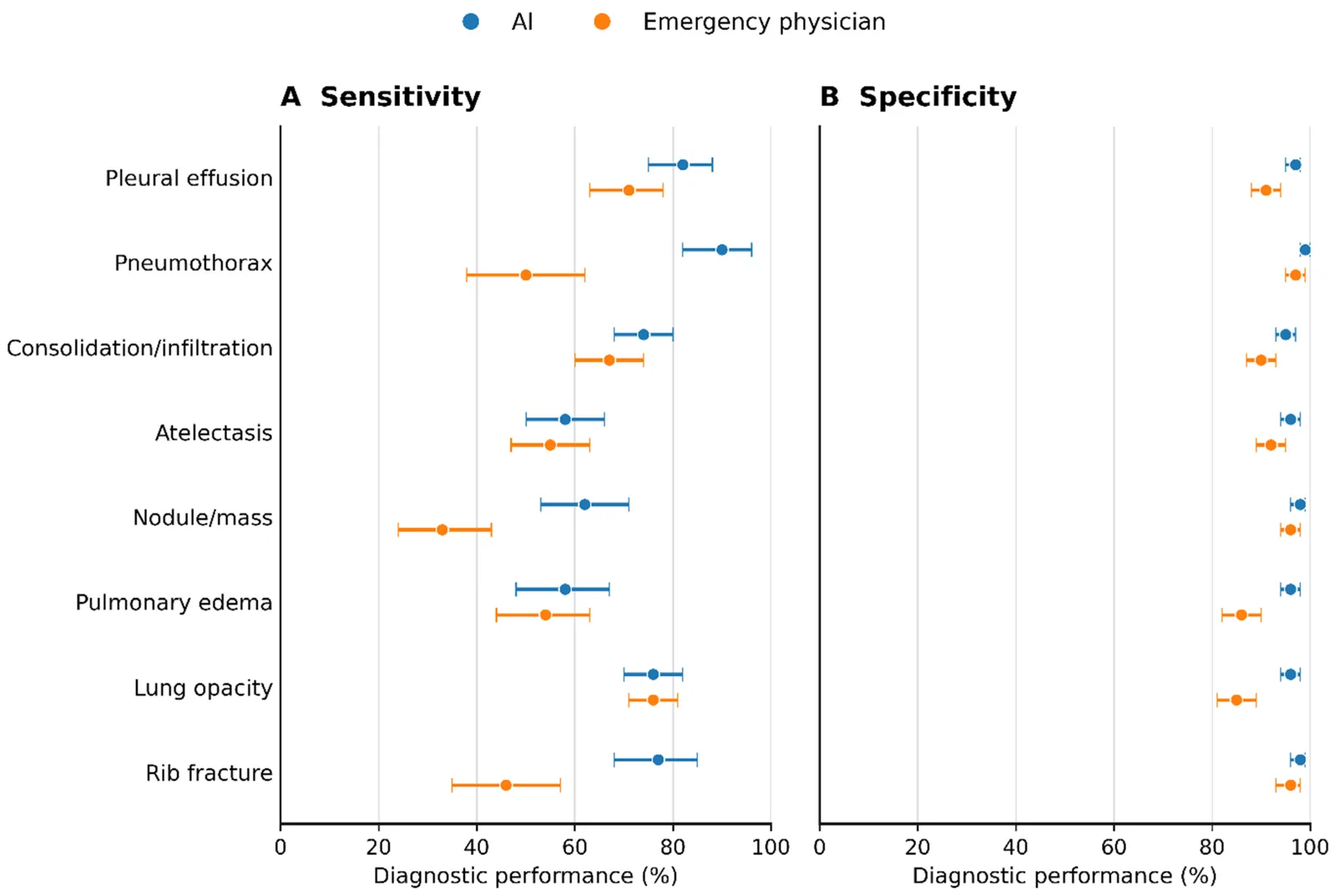
Target-specific sensitivity and specificity of AI and the emergency physician: (**A**) sensitivity and (**B**) specificity. Points are estimates, and horizontal bars are 95% Wilson confidence intervals. Exact target matching with the CT reference standard was used.

The most prevalent CT-confirmed targets were consolidation/infiltration and lung opacity (each n = 249; 27.1%), followed by pleural effusion (n = 217; 23.6%) and atelectasis (n = 153; 16.6%). Pneumothorax was least frequent (n = 65; 7.1%). AI sensitivity ranged from 58.0% for pulmonary edema to 90.8% for pneumothorax, whereas specificity ranged from 94.6% for consolidation/infiltration to 99.8% for pneumothorax. AI accuracy was ≥89.2% for every target and reached 99.1% for pneumothorax. Emergency physician sensitivity ranged from 32.7% for nodule/mass to 76.3% for lung opacity, with specificity ranging from 85.8% to 97.9%. The largest sensitivity differences favored AI for pneumothorax (90.8% [95% CI, 81.3–95.7] vs. 50.8% [38.9–62.5]), nodule/mass (63.3% [53.4–72.1] vs. 32.7% [24.2–42.4]), and rib fracture (77.2% [68.1–84.3] vs. 46.5% [37.1–56.2]) (Table 2; Figure 2).

In paired analyses, the AI–physician sensitivity differences remained significant after Holm correction for pneumothorax (+40.0 percentage points [95% CI, +24.6 to +55.4]), nodule/mass (+30.6 points [+16.3 to +43.9]), and rib fracture (+30.7 points [+17.8 to +43.6]; all adjusted *p* < 0.001). Sensitivity differences for the remaining targets were not significant after multiplicity adjustment. AI specificity was significantly higher for six targets, with adjusted differences ranging from +2.5 points for pneumothorax to +9.8 points for lung opacity; differences for nodule/mass and rib fracture were not significant after correction. AI accuracy was significantly higher across all eight targets, with absolute improvements of 4.2–8.2 percentage points (all adjusted *p* ≤ 0.001) (Table 3).

**Figure 3:**
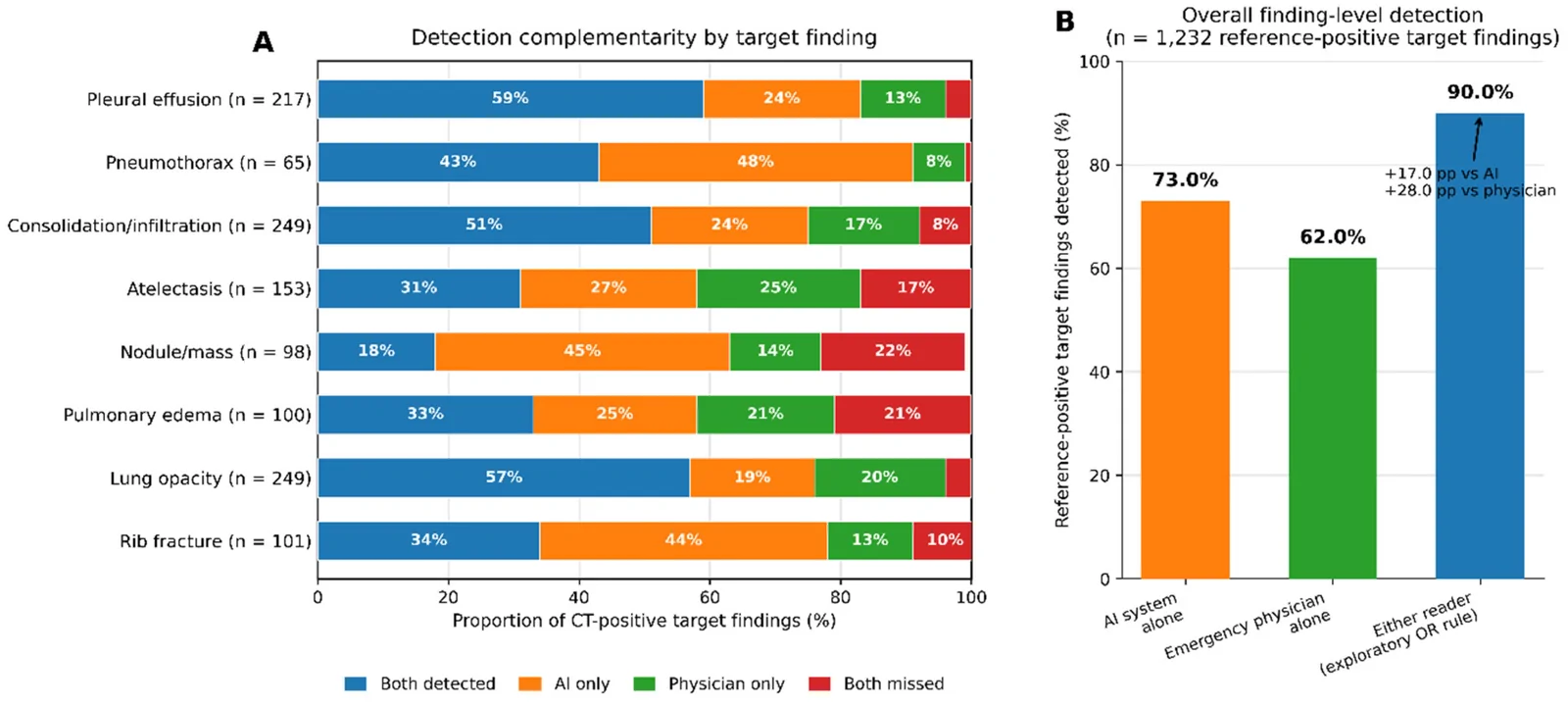
AI–physician complementarity for CT-confirmed findings. Panel (**A**) partitions each CT-positive target into both detected, AI only, physician only, and both missed. Panel (**B**) summarizes finding-level detection across 1232 CT-positive target assessments. The exploratory same-target OR rule detected 90.0%, an absolute increase of 17.0 percentage points over AI alone and 28.0 points over emergency physician interpretation alone.

Among CT-positive findings, both readers detected the same target in 18.4–58.5% of cases. AI-only detection was most prominent for pneumothorax (47.7%), nodule/mass (44.9%), and rib fracture (43.6%), whereas physician-only detection ranged from 7.7% for pneumothorax to 24.8% for atelectasis. The proportions missed by both readers were 1.5% for pneumothorax, 4.4% for lung opacity, and 5.1% for pleural effusion, but reached 22.4% for nodule/mass and 21.0% for pulmonary edema. The same-target OR rule achieved sensitivities of 77.6–98.5%, providing increments of 7.7–24.8 percentage points over AI alone and 19.3–47.7 points over physician interpretation alone, depending on the target (Table 4; Figure 3).

In the exploratory pooled analysis of 7360 patient–target assessments, AI achieved 73.0% sensitivity (cluster-bootstrap 95% CI, 70.5–75.4), 97.0% specificity (96.6–97.5), and 93.0% accuracy (92.4–93.6), compared with 62.0% (59.4–64.7), 92.5% (91.8–93.1), and 87.4% (86.6–88.1), respectively, for the emergency physician. Compared with emergency physician interpretation alone, the same-target OR rule increased pooled finding-level sensitivity from 62.0% to 90.0%, corresponding to an absolute gain of 28.0 percentage points. Relative to AI alone, sensitivity increased from 73.0% to 90.0%, an absolute gain of 17.0 percentage points (Table 5; Figure 3B). The OR rule also achieved an NPV of 97.8% (97.4–98.2), although specificity decreased to 90.0% (89.2–90.7). Conversely, the AND rule yielded 99.5% specificity (99.4–99.7) and 95.2% PPV (93.4–96.8) but reduced sensitivity to 45.0% (42.3–47.6) (Table 5). As a complementary descriptive analysis, we additionally computed macro-averaged performance, giving each of the eight targets equal weight regardless of prevalence (Table 5). Macro-averaged sensitivity was 72.5% (patient-level bootstrap 95% CI, 69.8–75.1) for AI and 56.8% (54.0–59.8) for the physician—a gap somewhat larger than the corresponding micro-averaged difference (73.0% vs. 62.0%) because the micro-average is weighted toward higher-prevalence targets (consolidation/infiltration, lung opacity) on which physician sensitivity was relatively preserved. Macro-averaged specificity (96.9% vs. 92.2%) and accuracy (93.0% vs. 87.4%) were materially similar to the micro-averaged values. Because both weighting schemes remain descriptive summaries across eight clinically heterogeneous targets, target-specific results (Table 2 and Table 3) remain the primary evidence.

**Figure 4:**
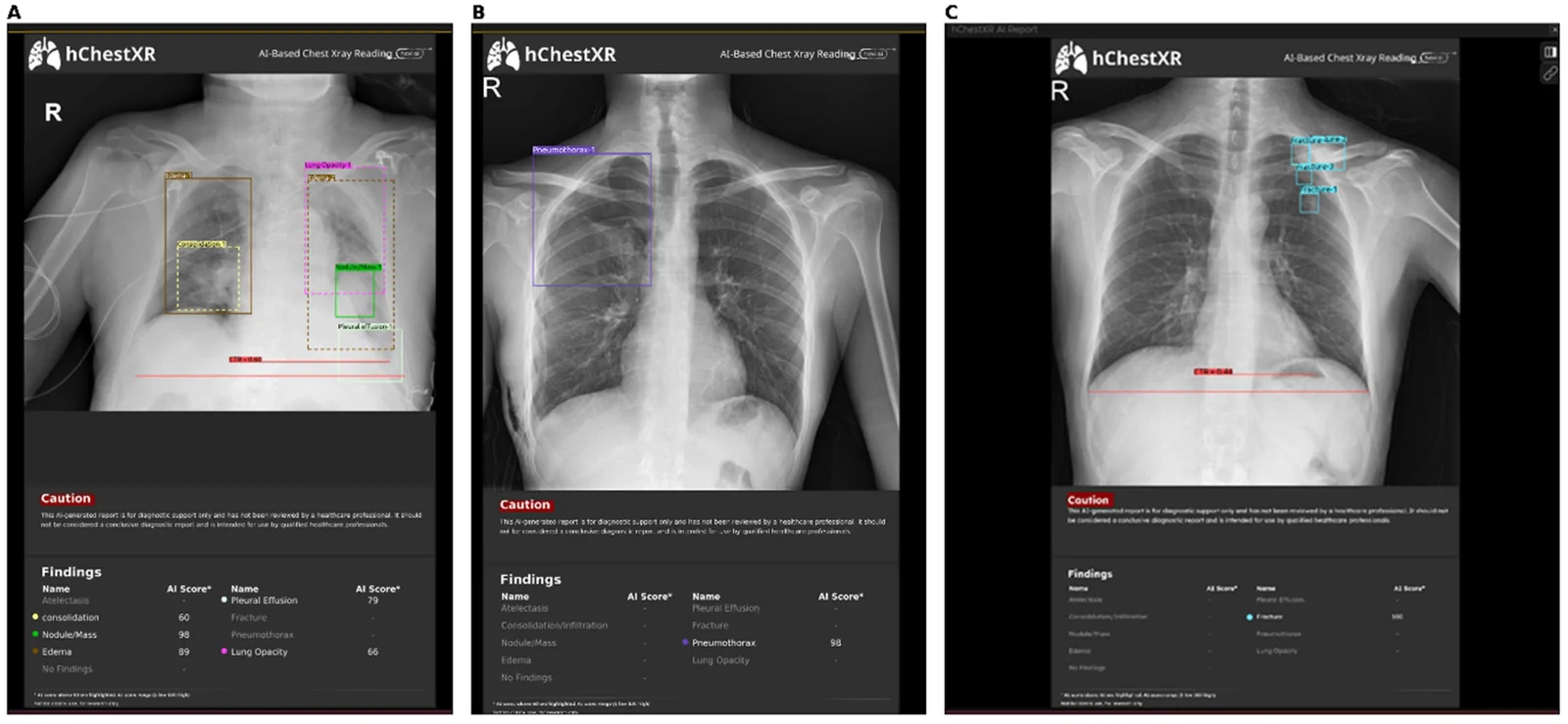
Representative examples of artificial intelligence–generated chest radiograph outputs. (**A**) Simultaneous detection and localization of multiple thoracic findings, including consolidation/infiltration, nodule/mass, pulmonary edema, pleural effusion, and lung opacity. (**B**) Detection and localization of a right-sided pneumothorax with a high AI confidence score. (**C**) Detection and localization of rib fractures. Colored bounding boxes indicate the regions highlighted by the AI system, and the accompanying scores represent the model’s 1–100 confidence values. These images are presented solely to illustrate the visual output of the AI platform and were not used as independent evidence of diagnostic performance. R, right.

Because predictive values are prevalence-dependent, the PPV and NPV reported in Table S1 apply specifically to this CT-selected, abnormality-enriched cohort (7.1–27.1% CT-positive across targets) and should not be extrapolated to an unselected emergency-department chest-radiograph population, in which lower pretest prevalence would be expected to reduce PPV and increase NPV for the same underlying sensitivity and specificity. Target-specific predictive values, likelihood ratios, F1 scores, and agreement estimates are reported in the supplementary analysis (Table S1). In the 769-patient subgroup with a CXR-to-CT interval ≤ 120 min, sensitivity and specificity estimates remained broadly consistent with the full-cohort findings (Table S2). Representative AI-generated localization outputs are presented for pleural and pulmonary parenchymal findings, pneumothorax, and rib fractures (Figure 4).

## 4. Discussion

The principal finding of this CT-referenced study was that artificial intelligence (AI) and the emergency physician differed not only in their overall diagnostic performance but also in the CT-referenced patterns of agreement and discordance across individual findings. AI identified a substantial proportion of CT-positive pneumothoraces, nodules/masses, and rib fractures not identified by the physician, whereas the emergency physician contributed additional detections of atelectasis, pulmonary edema, and lung opacity not identified by AI. This reciprocal but asymmetric complementarity became quantitatively apparent when the same-target OR rule increased pooled finding-level sensitivity from 62.0% for physician interpretation alone to 90.0%, an absolute gain of 28.0 percentage points; compared with the 73.0% sensitivity of AI alone, the gain was 17.0 points. However, the accompanying reduction in specificity to 90.0% indicates that the clinical value of complementarity must be considered together with the additional false-positive burden, rather than solely in terms of findings recovered (Table 4 and Table 5; Figure 3). Notably, 22.4% of CT-positive nodules/masses were missed by both readers, indicating that concordant negative AI-and-physician interpretation does not reliably exclude this finding in this cohort.

The main contribution of this study is not the identification of a single superior reader, but the determination of who detected each CT-positive target, and whether this complementarity varies systematically by abnormality type. Complementarity was bidirectional. AI-only detection accounted for 47.7% of pneumothoraces, 44.9% of nodules/masses, and 43.6% of rib fractures, whereas physician-only detection accounted for 24.8% of atelectases, 21.0% of pulmonary edema findings, and 19.7% of lung opacities. Multireader studies show that AI assistance may improve average performance, although the benefit varies by abnormality and reader [8,9,12,13]. Studies involving emergency and nonradiologist physicians also report improved accuracy with AI assistance, while demonstrating that erroneous suggestions may attenuate the benefit [14,15].

Two individual-patient cases illustrate this complementarity. In one patient (a 25-year-old man presenting with dyspnea, yellow triage), hChestXR flagged pneumothorax with a confidence score of 91/100 within 2 min of image acquisition, while the blinded physician read this radiograph as negative for pneumothorax; CT performed 71 min later confirmed an isolated pneumothorax, and the patient was admitted to the ward. Conversely, in another patient (a 58-year-old man presenting with cough, red triage), the blinded physician correctly identified atelectasis, which hChestXR did not flag (AI score ≤ 50); CT performed 49 min later confirmed isolated atelectasis, and the patient was admitted to the ward. These paired examples illustrate, at the individual-patient level, the bidirectional complementarity summarized statistically in Table 4.

Pneumothorax provided the clearest example of AI complementing physician interpretation. AI achieved 90.8% sensitivity and 99.8% specificity, whereas physician sensitivity was 50.8%; the 40.0-point sensitivity advantage remained significant after Holm adjustment. Hwang et al. demonstrated AI detection of important emergency thoracic abnormalities [1], and López Alcolea et al. reported stronger commercial-AI performance for pneumothorax than for several other targets [10]. Ahn et al. found notable AI-assisted gains for pneumothorax and nodules [16]. Novak et al. reported that assistance increased pneumothorax sensitivity among acute-care clinicians from 66.8% to 78.1%, especially in less experienced readers [17], while Hillis et al. demonstrated high standalone model performance [18]. Because commercial and multi-dataset evaluations show variation by institution, equipment, threshold, and case mix, our estimates apply specifically to hChestXR v1, PA radiographs, and this CT-verified emergency cohort [19,20].

Nodule/mass and rib fracture were the other targets with the largest AI contribution. AI sensitivity was 63.3% and 77.2%, respectively, compared with physician sensitivities of 32.7% and 46.5%; both differences were approximately 31 points. Multicenter and randomized nodule studies show that AI assistance can increase reader sensitivity [21,22], although incorrect recommendations may misdirect readers [23]. Huang et al. reported 74.5% AI sensitivity for rib fracture in 23,251 emergency radiographs, close to our 77.2% estimate [24]. Importantly, 22.4% of CT-positive nodules/masses were missed by both readers. Thus, AI’s incremental detections do not establish that concordant negative assessments safely exclude a nodule/mass or remove the need for clinically indicated CT.

Complementarity was more balanced for parenchymal and fluid-related findings. After multiplicity adjustment, sensitivity did not differ significantly for consolidation/infiltration, atelectasis, pulmonary edema, or lung opacity; opacity sensitivities were nearly identical at 75.9% and 76.3%. The physician-only detections may reflect complementary recognition of parenchymal and fluid-related changes, although lesion morphology was not directly analyzed. Target-dependent discrepancies between emergency physicians and radiologists are recognized [2], while paired CXR–CT studies show that CT reveals more small parenchymal and pleural findings [3,25]. CT therefore created a more demanding but anatomically more sensitive reference than the original radiograph report. Target-level heterogeneity in recent emergency and commercial-AI evaluations further supports this interpretation [19,20,26].

The pooled analysis demonstrates both the magnitude and sensitivity–specificity trade-off of complementarity. The OR rule increased sensitivity to 90.0% and NPV to 97.8%, but reduced specificity from 97.0% for AI alone to 90.0%. The AND rule achieved 99.5% specificity and 95.2% PPV, while lowering sensitivity to 45.0%. A sensitivity-oriented alert strategy and high-certainty confirmation therefore cannot be achieved through the same rule. Moreover, these were statistical simulations of independent decisions, not clinical interventions. Automation bias, experience, trust, and incorrect AI suggestions may alter actual human–AI performance [12,13,14]. The study consequently identifies a complementarity signal for prospective testing; it does not establish that AI improved this physician’s performance. The clinical consequences of a false negative and a false positive are not symmetric: a missed pneumothorax or a missed nodule/mass carries materially greater downstream risk than an unnecessary confirmatory review triggered by a false-positive alert. This asymmetry is relevant when weighing the sensitivity gain and specificity loss produced by the simulated OR rule (Table 5) and should inform how any future AI-alerting threshold is chosen for clinical deployment, rather than optimizing a single combined accuracy metric. How discordant AI-and-physician interpretations should be adjudicated in practice—for example, which combination should trigger mandatory second review—is an operational question for prospective workflow studies rather than one this retrospective paired-accuracy design can resolve.

An additional interpretive caveat concerns an assumption implicit in any CT-referenced accuracy study: a CT-positive finding is not necessarily radiographically detectable. Small pneumothoraces, subtle pleural effusions, small nodules, and non-displaced rib fractures can be confidently identified on CT while remaining genuinely occult on a two-dimensional PA radiograph. Lesion size, extent, and clinical severity were not systematically extracted from the CT reports in this study, so we cannot directly stratify the reported false-negative and ‘both-missed’ proportions by radiographic detectability. Consequently, some of the CT-positive/CXR-negative classifications attributed to the physician (and, less often, to AI) in Table 4 may reflect the intrinsic sensitivity limits of chest radiography rather than a genuine interpretive error, particularly for pneumothorax, nodule/mass, and rib fracture—the three targets with the largest AI–physician sensitivity gaps. We recommend that future prospective work capture lesion size or extent alongside the binary reference classification used here.

The median 4 min interval between radiograph acquisition and AI output suggests that complementary information could be available during initial assessment. Previous studies indicate that AI may prioritize critical radiographs, improve sensitivity for important emergency findings, and shorten interpretation time [7,26,27]. Our measurement represents system turnaround, not reading time. Because treatment, management changes, length of stay, and outcomes were not evaluated causally, rapid output is not evidence of clinical or economic benefit.

The broadly similar results in the 769 patients with a CXR-to-CT interval of ≤120 min support the robustness of the principal performance pattern to a shorter temporal window. Unavailable raw scores for AI outputs ≤ 50 prevented ROC, calibration, and alternative-threshold analyses; consequently, we cannot determine whether the sensitivity–specificity balance reported here is intrinsic to the model or an artifact of this single fixed operating point. Manufacturer thresholds may produce different sensitivity–alert burden trade-offs across targets and subgroups [28]; our estimates therefore apply to the predefined >50 operating point, which should be explicitly reported under STARD-AI [11].

From a clinical imaging perspective, these findings support prospective evaluation of AI as a rapid second-reader tool, particularly for targets with substantial AI-only detection. However, the gain in sensitivity with the simulated OR strategy was accompanied by reduced specificity, highlighting the potential alert burden of sensitivity-oriented deployment. Because the combination reflected independent readings rather than real-time AI-assisted interpretation, future multireader studies should evaluate actual workflow effects, downstream imaging, treatment decisions, and patient-centered outcomes.

## 5. Strengths and Limitations

This study has several strengths. Consecutive eligible patients were included without sampling, and the paired design allowed AI and emergency-physician interpretations to be compared in the same patients against the same CT reference standard. Exact target matching reduced misclassification across findings. Additional strengths included blinded and randomized physician assessment, use of an unchanged commercial AI version at the manufacturer-defined threshold, a prespecified 4 h CXR-to-CT window, exclusion of image-altering interventions, adjudication of uncertain CT reports, and a ≤120 min sensitivity analysis. Patient-level bootstrap methods, cluster resampling, and Holm adjustment addressed within-patient dependency and multiple comparisons.

Several limitations should be considered. First, the retrospective design and simulated OR/AND rules do not establish that AI assistance improves physician performance, clinical decisions, or patient outcomes. Second, radiographs were interpreted by a single emergency physician without clinical information or prior imaging, limiting assessment of interreader variability and potentially underestimating real-world physician performance. Third, the single-center, CT-selected cohort limits generalizability and introduces potential verification bias because AI outputs available in routine PACS may have influenced the decision to obtain CT. Results therefore may not apply to unselected emergency populations or portable AP radiographs. Finally, the CT reference standard relied mainly on routine radiology reports rather than centralized rereading, raw AI scores ≤ 50 were unavailable, and no geographic external validation or powered subgroup analyses were performed. Radiograph technical quality (inspiration effort, rotation, positioning, and penetration) was not systematically graded and could not be analyzed as an effect modifier; restricting inclusion to standardized posteroanterior technique and excluding portable anteroposterior radiographs partially, but not completely, controls for this source of variability. Complete-case exclusion of patients with failed AI processing (n = 19) could itself introduce bias if processing failure is non-random with respect to image quality or pathology, which cannot be ruled out retrospectively. Subgroup analyses by patient characteristics were not performed because several targets already have as few as 65–101 CT-positive cases, and further subdivision would be underpowered and prone to spurious findings; this is deferred to a larger, prospective, multicenter design. The structured research dataset did not include the denominator of all adult PA chest radiographs performed during the study period; therefore, a reliable CT-verification proportion could not be calculated, and these diagnostic-performance estimates should be understood to describe a CT-selected subgroup rather than routine chest radiography practice. Hevi AI (Istanbul, Türkiye), the manufacturer of hChestXR, describes the product as a deep-learning system assessing common thoracic radiographic findings, and the product is listed with CE regulatory marking on at least one third-party clinical-AI marketplace [29]; however, granular architectural details, training-dataset composition, and internal validation methodology are proprietary to the manufacturer and were not accessible to the investigators, so our evaluation is an external, real-world performance assessment of a fixed commercial product rather than a technical validation of its underlying model. Additionally, the generic AI ‘fracture’ output was evaluated against CT-confirmed rib fracture, while non-rib thoracic fractures were not systematically coded; therefore, some apparent false-positive AI fracture classifications may reflect this target-mapping mismatch. Certainty terminology in the original CT reports was not retained as a separate structured variable, precluding a sensitivity analysis restricted to definite CT-positive findings. Accordingly, the findings apply primarily to hChestXR version 1 at the manufacturer-defined >50 threshold in this CT-selected PA cohort.

## 6. Conclusions

In this CT-referenced emergency cohort, the AI system and the single study emergency-physician reader demonstrated target-dependent, partly non-overlapping strengths. AI contributed most to pneumothorax, nodule/mass, and rib fracture detection, whereas physician-only detections were more frequent for atelectasis, pulmonary edema, and lung opacity. Exploratory simulated OR/AND analyses demonstrated a sensitivity–specificity trade-off across pooled patient–target assessments but did not represent observed AI-assisted clinical performance. These findings demonstrate potential diagnostic complementarity rather than proven benefit from AI-assisted care. These findings are specific to hChestXR version 1, this single-center CT-selected cohort, and predominantly PA radiographs, and should not be assumed to generalize to other commercial AI systems, institutions, or acquisition protocols without external validation. A prospective, multicenter, multireader crossover study is needed to determine whether displaying AI outputs improves emergency-physician diagnostic decisions and clinically relevant outcomes.

## Figures and Tables

**Table 1:** Clinical, workflow, and disposition characteristics of the study cohort.

Characteristic	Overall (N = 920)
Age, years, mean ± SD	55.0 ± 18.4
**Sex**	
Male	479 (52.1%)
Female	441 (47.9%)
**Chief complaint**	
Trauma	273 (29.7%)
Dyspnea	238 (25.9%)
Chest pain	158 (17.2%)
Cough	115 (12.5%)
Fever	83 (9.0%)
Altered mental status	32 (3.5%)
Other	21 (2.3%)
**Triage category**	
Red	178 (19.3%)
Yellow	510 (55.4%)
Green	232 (25.2%)
Emergency department length of stay, min, median [IQR]	242.0 [154.0–331.5]
AI report turnaround, min, median [IQR]	4.0 [3.0–6.0]
CXR-to-CT interval, min, median [IQR]	73.0 [51.0–100.0]
CT report turnaround, min, median [IQR]	146.0 [116.0–193.0]
**Disposition**	
Discharged	487 (52.9%)
Ward admission	282 (30.7%)
Intensive care admission	99 (10.8%)
Transfer	44 (4.8%)
Death	8 (0.9%)
At least one CT-confirmed target finding	689 (74.9%)
No CT-confirmed target finding	231 (25.1%)

Data are presented as n (%), mean ± SD, or median [IQR], as appropriate. Bold text indicates category headings. AI, artificial intelligence; CT, computed tomography; CXR, chest radiograph; IQR, interquartile range; SD, standard deviation. The patient-level presence of any CT target is descriptive and was not used as a diagnostic accuracy endpoint.

**Table 2:** CT reference-standard prevalence and target-specific diagnostic performance.

Target Finding	CT Positive, n (%)	Reader	TP	FP	TN	FN	Sensitivity (95% CI)	Specificity (95% CI)	Accuracy (95% CI)
Pleural effusion	217 (23.6%)	AI	178	30	673	39	82.0% (76.4–86.6)	95.7% (94.0–97.0)	92.5% (90.6–94.0)
Pleural effusion	217 (23.6%)	Emergency physician	155	59	644	62	71.4% (65.1–77.0)	91.6% (89.3–93.4)	86.8% (84.5–88.9)
Pneumothorax	65 (7.1%)	AI	59	2	853	6	90.8% (81.3–95.7)	99.8% (99.2–99.9)	99.1% (98.3–99.6)
Pneumothorax	65 (7.1%)	Emergency physician	33	23	832	32	50.8% (38.9–62.5)	97.3% (96.0–98.2)	94.0% (92.3–95.4)
Consolidation/ infiltration	249 (27.1%)	AI	186	36	635	63	74.7% (69.0–79.7)	94.6% (92.7–96.1)	89.2% (87.1–91.1)
Consolidation/ infiltration	249 (27.1%)	Emergency physician	168	68	603	81	67.5% (61.4–73.0)	89.9% (87.4–91.9)	83.8% (81.3–86.0)
Atelectasis	153 (16.6%)	AI	89	29	738	64	58.2% (50.2–65.7)	96.2% (94.6–97.4)	89.9% (87.8–91.7)
Atelectasis	153 (16.6%)	Emergency physician	85	69	698	68	55.6% (47.6–63.2)	91.0% (88.8–92.8)	85.1% (82.7–87.3)
Nodule/mass	98 (10.7%)	AI	62	11	811	36	63.3% (53.4–72.1)	98.7% (97.6–99.3)	94.9% (93.3–96.1)
Nodule/mass	98 (10.7%)	Emergency physician	32	25	797	66	32.7% (24.2–42.4)	97.0% (95.5–97.9)	90.1% (88.0–91.9)
Pulmonary edema	100 (10.9%)	AI	58	35	785	42	58.0% (48.2–67.2)	95.7% (94.1–96.9)	91.6% (89.7–93.3)
Pulmonary edema	100 (10.9%)	Emergency physician	54	106	714	46	54.0% (44.3–63.4)	87.1% (84.6–89.2)	83.5% (80.9–85.7)
Lung opacity	249 (27.1%)	AI	189	29	642	60	75.9% (70.2–80.8)	95.7% (93.9–97.0)	90.3% (88.2–92.1)
Lung opacity	249 (27.1%)	Emergency physician	190	95	576	59	76.3% (70.6–81.2)	85.8% (83.0–88.3)	83.3% (80.7–85.5)
Rib fracture	101 (11.0%)	AI	78	9	810	23	77.2% (68.1–84.3)	98.9% (97.9–99.4)	96.5% (95.1–97.5)
Rib fracture	101 (11.0%)	Emergency physician	47	17	802	54	46.5% (37.1–56.2)	97.9% (96.7–98.7)	92.3% (90.4–93.8)

Each target was analyzed as a separate binary diagnostic task, and each AI and emergency-physician classification was compared only with the corresponding CT finding. CI, confidence interval; FN, false negative; FP, false positive; TN, true negative; TP, true positive. Confidence intervals for proportions are Wilson 95% CIs.

**Table 3:** Paired comparison of AI and emergency physician diagnostic performance.

Target Finding	Sensitivity			Specificity			Accuracy		
	Difference, pp (95% CI)	Exact *p*	Adj. *p*	Difference, pp (95% CI)	Exact *p*	Adj. *p*	Difference, pp (95% CI)	Exact *p*	Adj. *p*
Pleural effusion	+10.6 pp (+2.8 to +18.4)	0.013	0.064	+4.1 pp (+2.0 to +6.3)	<0.001	**0.001**	+5.7 pp (+3.2 to +8.2)	<0.001	**<0.001**
Pneumothorax	+40.0 pp (+24.6 to +55.4)	<0.001	**<0.001**	+2.5 pp (+1.4 to +3.6)	<0.001	**<0.001**	+5.1 pp (+3.6 to +6.7)	<0.001	**<0.001**
Consolidation/infiltration	+7.2 pp (−0.8 to +15.3)	0.092	0.367	+4.8 pp (+1.9 to +7.6)	0.002	**0.005**	+5.4 pp (+2.4 to +8.4)	<0.001	**<0.001**
Atelectasis	+2.6 pp (−8.5 to +13.7)	0.738	1.000	+5.2 pp (+2.9 to +7.6)	<0.001	**<0.001**	+4.8 pp (+2.0 to +7.5)	0.001	**0.001**
Nodule/mass	+30.6 pp (+16.3 to +43.9)	<0.001	**<0.001**	+1.7 pp (+0.2 to +3.2)	0.029	0.058	+4.8 pp (+2.7 to +6.8)	<0.001	**<0.001**
Pulmonary edema	+4.0 pp (−9.0 to +17.0)	0.659	1.000	+8.7 pp (+6.1 to +11.2)	<0.001	**<0.001**	+8.2 pp (+5.3 to +11.0)	<0.001	**<0.001**
Lung opacity	−0.4 pp (−8.0 to +7.2)	1.000	1.000	+9.8 pp (+6.9 to +13.0)	<0.001	**<0.001**	+7.1 pp (+4.0 to +10.1)	<0.001	**<0.001**
Rib fracture	+30.7 pp (+17.8 to +43.6)	<0.001	**<0.001**	+1.0 pp (−0.2 to +2.2)	0.169	0.169	+4.2 pp (+2.3 to +6.1)	<0.001	**<0.001**

Values are absolute paired differences (AI minus emergency physician) in percentage points with unadjusted patient-level bootstrap 95% CIs (10,000 resamples). Exact two-sided McNemar *p* values are reported. Holm adjustment was applied separately across the eight targets for sensitivity, specificity, and accuracy. Bold indicates Holm-adjusted *p* < 0.05. Adj. *p*, Holm-adjusted *p*-value.

**Table 4:** Exact-target AI–physician complementarity and incremental sensitivity.

Target Finding	CT+, n	Both Detected, n (%)	AI Only, n (%)	Physician Only, n (%)	Both Missed, n (%)	OR Sensitivity (95% CI)	OR Specificity (95% CI)	Increment vs. AI, pp (95% CI)	Increment vs. Physician, pp (95% CI)
Pleural effusion	217	127 (58.5%)	51 (23.5%)	28 (12.9%)	11 (5.1%)	94.9% (91.2–97.1)	89.2% (86.7–91.3)	+12.9 (9.1–18.0)	+23.5 (18.4–29.6)
Pneumothorax	65	28 (43.1%)	31 (47.7%)	5 (7.7%)	1 (1.5%)	98.5% (91.8–99.7)	97.1% (95.7–98.0)	+7.7 (3.3–16.8)	+47.7 (36.0–59.6)
Consolidation/ infiltration	249	126 (50.6%)	60 (24.1%)	42 (16.9%)	21 (8.4%)	91.6% (87.5–94.4)	84.9% (82.0–87.5)	+16.9 (12.7–22.0)	+24.1 (19.2–29.8)
Atelectasis	153	47 (30.7%)	42 (27.5%)	38 (24.8%)	26 (17.0%)	83.0% (76.3–88.1)	87.5% (85.0–89.6)	+24.8 (18.7–32.2)	+27.5 (21.0–35.0)
Nodule/mass	98	18 (18.4%)	44 (44.9%)	14 (14.3%)	22 (22.4%)	77.6% (68.3–84.7)	95.6% (94.0–96.8)	+14.3 (8.7–22.6)	+44.9 (35.4–54.8)
Pulmonary edema	100	33 (33.0%)	25 (25.0%)	21 (21.0%)	21 (21.0%)	79.0% (70.0–85.8)	83.5% (80.8–85.9)	+21.0 (14.2–30.0)	+25.0 (17.5–34.3)
Lung opacity	249	141 (56.6%)	48 (19.3%)	49 (19.7%)	11 (4.4%)	95.6% (92.3–97.5)	82.1% (79.0–84.8)	+19.7 (15.2–25.1)	+19.3 (14.9–24.6)
Rib fracture	101	34 (33.7%)	44 (43.6%)	13 (12.9%)	10 (9.9%)	90.1% (82.7–94.5)	96.8% (95.4–97.8)	+12.9 (7.7–20.8)	+43.6 (34.3–53.3)

Complementarity categories use CT-positive cases for the same target as the denominator and are mutually exclusive. The same-target OR rule was positive when either reader identified that target. Increment versus AI equals the physician-only proportion; increment versus physician equals the AI-only proportion. Increment confidence intervals are Wilson 95% CIs. The OR analysis is exploratory, and its specificity is reported to show the sensitivity–specificity trade-off. The both-detected/AI-only/physician-only/both-missed breakdown constitutes a four-way paired classification of AI and physician outcomes for each target.

**Table 5:** Exploratory pooled (micro-averaged) and complementary macro-averaged diagnostic performance across 7360 patient–target assessments.

Method	TP/FP/TN/FN	Sensitivity (Cluster-Bootstrap 95% CI)	Specificity (Cluster-Bootstrap 95% CI)	PPV (Cluster-Bootstrap 95% CI)	NPV (Cluster-Bootstrap 95% CI)	Accuracy (Cluster-Bootstrap 95% CI)
AI	899/181/5947/333	73.0% (70.5–75.4)	97.0% (96.6–97.5)	83.2% (80.9–85.5)	94.7% (94.1–95.2)	93.0% (92.4–93.6)
Emergency physician	764/462/5666/468	62.0% (59.4–64.7)	92.5% (91.8–93.1)	62.3% (59.6–65.0)	92.4% (91.7–93.1)	87.4% (86.6–88.1)
Same-target OR (simulated)	1109/615/5513/123	90.0% (88.3–91.7)	90.0% (89.2–90.7)	64.3% (62.0–66.6)	97.8% (97.4–98.2)	90.0% (89.3–90.6)
Same-target AND (simulated)	554/28/6100/678	45.0% (42.3–47.6)	99.5% (99.4–99.7)	95.2% (93.4–96.8)	90.0% (89.3–90.7)	90.4% (89.7–91.1)
AI (macro-averaged)	—	72.5% (69.8–75.1)	96.9% (96.5–97.3)	—	—	93.0% (92.4–93.6)
Physician (macro-averaged)	—	56.8% (54.0–59.8)	92.2% (91.5–92.9)	—	—	87.4% (86.6–88.1)

Patient-cluster bootstrap resampling (10,000 replicates) retained all eight target observations from each sampled patient. Because targets differ in prevalence and diagnostic difficulty, these pooled results are supportive and target-specific estimates remain primary. OR, either reader positive for the same target; AND, both readers positive for the same target. Macro-averaged rows give each target equal weight (unweighted mean of the eight target-specific estimates, Table 2) using the same patient-level bootstrap methodology; TP/FP/TN/FN, PPV, and NPV are not meaningfully defined under equal target-weighting because predictive values depend on each target’s prevalence, and are therefore not reported (—) for these two rows.

## Data Availability

The deidentified data supporting the findings are available from the corresponding author upon reasonable request, subject to institutional and ethics approval and applicable patient-privacy regulations.

## Supplementary Materials

The following supporting information can be downloaded at https://www.mdpi.com/article/10.3390/tomography12100142/s1. Table S1: Extended target-specific diagnostic performance; Table S2: Robustness analysis restricted to a CXR-to-CT interval of ≤120 min (n = 769); Table S3: Target-by-target harmonization of AI, physician, and CT reference-standard definitions.
