# Supplementary material for: CT-Referenced Diagnostic Performance and Complementary Error Patterns of Artificial Intelligence Versus a Single Emergency Physician in Chest Radiograph Interpretation: A Retrospective Paired Diagnostic Accuracy Study
Source: Tomography. 2026 Oct 1;12(10):142. doi: 10.3390/tomography12100142 (PMC13709469; doi:10.3390/tomography12100142)
Supplement: Supplementary file 1 [file tomography-12-00142-s001.zip › tomography-4556072-supplementary.pdf]

Supplementary Materials

Table S1. Extended target-specific diagnostic performance.

Target finding	Reader	PPV (95% CI)	NPV (95% CI)	LR+ (95% CI)	LR- (95% CI)	F1 score	Cohen kappa
Pleural effusion	AI	85.6% (80.2–89.7)	94.5% (92.6–96.0)	19.22 (13.47–27.43)	0.19 (0.14–0.25)	0.838	0.789
Pleural effusion	Emergency physician	72.4% (66.1–78.0)	91.2% (88.9–93.1)	8.51 (6.57–11.02)	0.31 (0.25–0.39)	0.719	0.633
Pneumothorax	AI	96.7% (88.8–99.1)	99.3% (98.5–99.7)	388 (96.99–1552.4)	0.09 (0.04–0.20)	0.937	0.932
Pneumothorax	Emergency physician	58.9% (45.9–70.8)	96.3% (94.8–97.4)	18.87 (11.81–30.16)	0.51 (0.40–0.65)	0.545	0.514
Consolidation/infiltration	AI	83.8% (78.4–88.1)	91.0% (88.6–92.9)	13.92 (10.05–19.29)	0.27 (0.22–0.33)	0.790	0.718
Consolidation/infiltration	Emergency physician	71.2% (65.1–76.6)	88.2% (85.5–90.4)	6.66 (5.23–8.47)	0.36 (0.30–0.43)	0.693	0.583
Atelectasis	AI	75.4% (66.9–82.3)	92.0% (89.9–93.7)	15.38 (10.51–22.53)	0.43 (0.36–0.52)	0.657	0.599
Atelectasis	Emergency physician	55.2% (47.3–62.8)	91.1% (88.9–92.9)	6.18 (4.73–8.06)	0.49 (0.41–0.58)	0.554	0.464
Nodule/mass	AI	84.9% (75.0–91.4)	95.7% (94.2–96.9)	47.28 (25.79–86.67)	0.37 (0.29–0.48)	0.725	0.698
Nodule/mass	Emergency physician	56.1% (43.3–68.2)	92.4% (90.4–93.9)	10.74 (6.65–17.34)	0.69 (0.60–0.80)	0.413	0.363
Pulmonary edema	AI	62.4% (52.2–71.5)	94.9% (93.2–96.2)	13.59 (9.44–19.57)	0.44 (0.35–0.55)	0.601	0.554
Pulmonary edema	Emergency physician	33.8% (26.9–41.4)	93.9% (92.0–95.4)	4.18 (3.24–5.38)	0.53 (0.43–0.65)	0.415	0.325
Lung opacity	AI	86.7% (81.5–90.6)	91.5% (89.2–93.3)	17.56 (12.22–25.24)	0.25 (0.20–0.31)	0.809	0.745
Lung opacity	Emergency physician	66.7% (61.0–71.9)	90.7% (88.2–92.7)	5.39 (4.42–6.57)	0.28 (0.22–0.35)	0.712	0.594
Rib fracture	AI	89.7% (81.5–94.5)	97.2% (95.9–98.2)	70.28 (36.38–135.74)	0.23 (0.16–0.33)	0.830	0.811
Rib fracture	Emergency physician	73.4% (61.5–82.7)	93.7% (91.9–95.1)	22.42 (13.40–37.51)	0.55 (0.46–0.66)	0.570	0.530

PPV, positive predictive value; NPV, negative predictive value; LR+, positive likelihood ratio; LR−, negative likelihood ratio. Cohen's κ describes agreement of each reader with the binary CT reference for the same target and is shown without a confidence interval. The very high AI LR+ for pneumothorax reflects only two false-positive AI classifications and should be interpreted together with its wide 95% CI.

Table S2. Robustness analysis restricted to a CXR-to-CT interval of ≤120 min (n = 769).

Target finding	Reader	Sensitivity, full cohort	Sensitivity, ≤120-min subset	Specificity, full cohort	Specificity, ≤120-min subset
Pleural effusion	AI	82.0% (76.4–86.6)	83.3% (77.3–88.0)	95.7% (94.0–97.0)	95.4% (93.3–96.8)
Pleural effusion	Emergency physician	71.4% (65.1–77.0)	69.9% (63.0–76.0)	91.6% (89.3–93.4)	91.6% (89.1–93.6)
Pneumothorax	AI	90.8% (81.3–95.7)	89.8% (79.5–95.3)	99.8% (99.2–99.9)	99.7% (99.0–99.9)
Pneumothorax	Emergency physician	50.8% (38.9–62.5)	49.2% (36.8–61.6)	97.3% (96.0–98.2)	97.0% (95.5–98.1)
Consolidation/infiltration	AI	74.7% (69.0–79.7)	74.0% (67.4–79.6)	94.6% (92.7–96.1)	93.9% (91.6–95.6)
Consolidation/infiltration	Emergency physician	67.5% (61.4–73.0)	67.9% (61.0–74.0)	89.9% (87.4–91.9)	90.1% (87.3–92.2)
Atelectasis	AI	58.2% (50.2–65.7)	62.0% (53.1–70.1)	96.2% (94.6–97.4)	97.2% (95.7–98.2)
Atelectasis	Emergency physician	55.6% (47.6–63.2)	52.9% (44.0–61.6)	91.0% (88.8–92.8)	90.4% (87.9–92.5)
Nodule/mass	AI	63.3% (53.4–72.1)	60.7% (50.0–70.5)	98.7% (97.6–99.3)	98.8% (97.7–99.4)
Nodule/mass	Emergency physician	32.7% (24.2–42.4)	31.0% (22.1–41.5)	97.0% (95.5–97.9)	96.9% (95.4–98.0)
Pulmonary edema	AI	58.0% (48.2–67.2)	55.0% (44.1–65.4)	95.7% (94.1–96.9)	96.5% (94.9–97.6)
Pulmonary edema	Emergency physician	54.0% (44.3–63.4)	53.8% (42.9–64.3)	87.1% (84.6–89.2)	87.1% (84.4–89.4)
Lung opacity	AI	75.9% (70.2–80.8)	74.8% (68.5–80.2)	95.7% (93.9–97.0)	95.5% (93.5–97.0)
Lung opacity	Emergency physician	76.3% (70.6–81.2)	74.8% (68.5–80.2)	85.8% (83.0–88.3)	85.2% (82.0–87.9)
Rib fracture	AI	77.2% (68.1–84.3)	76.4% (66.6–84.0)	98.9% (97.9–99.4)	98.8% (97.7–99.4)
Rib fracture	Emergency physician	46.5% (37.1–56.2)	47.2% (37.2–57.5)	97.9% (96.7–98.7)	97.9% (96.6–98.8)

The restricted analysis included 769 patients and used Wilson 95% CIs. The prespecified primary CXR-to-CT window was ≤4 h. The tighter ≤120-min analysis evaluates robustness to potential temporal evolution between radiography and CT; estimates were materially consistent with the full-cohort results.

Table S3. Target-by-target harmonization of AI, physician, and CT reference-standard definitions.

AI target label	Physician / CT target label	CT reference-standard definition	Included CT terminology / synonyms	Target-specific matching notes
Pleural effusion	Pleural effusion	Any pleural fluid collection reported on CT, any laterality/volume	"pleural effusion," "hemothorax" (mapped here), "hydrothorax"	Hemothorax was coded as pleural effusion because neither the AI output nor the physician reading form distinguishes fluid composition.
Pneumothorax	Pneumothorax	Any intrapleural air collection reported on CT	"pneumothorax"; air component of "hydropneumothorax"	—
Consolidation/infiltration	Consolidation/infiltration	Airspace disease reported as consolidation, infiltrate, or attributed to pneumonia or contusion	"consolidation," "infiltrate," "pneumonia," "contusion"	Pneumonia and pulmonary contusion were both mapped here because both are airspace-filling processes and the AI model outputs a single "consolidation/infiltration" category.
Atelectasis	Atelectasis	Volume loss/collapse reported as atelectasis	"atelectasis," "collapse," "subsegmental atelectasis"	—
Nodule/mass	Nodule/mass	Discrete pulmonary nodule or mass reported on CT	"nodule," "mass," "nodular opacity"	—
Pulmonary edema	Pulmonary edema	Interstitial or airspace edema reported on CT	"pulmonary edema," "interstitial edema," "cardiogenic edema pattern"	—
Lung opacity	Lung opacity	Parenchymal opacity explicitly described as such in the CT report; targets were not mutually exclusive, and co-existing consolidation/infiltration did not preclude lung-opacity coding when both were explicitly reported	"opacity"; unqualified "ground-glass opacity"	In this cohort, 70/920 patients (7.6%) were positive for both consolidation/infiltration and lung opacity simultaneously, when the CT report used both terms explicitly (28.1% of consolidation/infiltration-positive patients were also lung-opacity-positive), confirming the two labels were not simply redundant despite this overlap.

AI target label	Physician / CT target label	CT reference-standard definition	Included CT terminology / synonyms	Target-specific matching notes
Fracture	Rib fracture	CT-confirmed rib fracture specifically	“rib fracture”	As specified among the eight prespecified AI targets, this output was labeled generically as “fracture” rather than “rib fracture”; it was matched here only against CT-confirmed rib fracture. 9/920 patients had an AI-positive “fracture” call without a CT-confirmed rib fracture and were scored as false positives for this target; because non-rib thoracic fractures were not systematically coded, we could not determine how many of these nine represented genuine detections of a different thoracic bone injury. This target-mapping mismatch is acknowledged directly as a limitation rather than quantitatively bounded.

This table expands the abbreviated mappings given in the Methods (“CT reference standard and target harmonization”).
